# Preservation of the left colic artery in modified laparoscopic anterior rectal resections without auxiliary abdominal incisions for transanal specimen retrieval

**DOI:** 10.1186/s12893-022-01593-0

**Published:** 2022-04-21

**Authors:** Yulin Liu, Peng Yu, Han Li, Lijian Xia, Xiangmin Li, Meijuan Zhang, Zhonghui Cui, Jingbo Chen

**Affiliations:** 1 Department of General Surgery, Key Laboratory of Metabolism and Gastrointestinal Tumor, Key Laboratory of Laparoscopic Technology, Shandong Medicine and Health Key Laboratory of General Surgery, The First Affiliated Hospital of Shandong First Medical University & Shandong Provincial Qianfoshan Hospital, Jinan, People’s Republic of China; 2 Department of Gastrointestinal Surgery, The Second People’s Hospital of Lianyungang, Liaocheng, China; 3 Department of General Surgery, The First Affiliated Hospital of Shandong First Medical University & Shandong Provincial Qianfoshan Hospital, 16766 Jingshi Road, Jinan, 250014 Shandong People’s Republic of China

**Keywords:** Rectal malignant tumour, Natural orifice specimen extraction surgery, Preservation of left colic artery, Laparoscopy

## Abstract

**Background:**

Laparoscopic low anterior rectal resection is the most widely used surgical procedure for middle and low rectal cancer. The aim of this study was to investigate the feasibility and safety of the extracorporeal placement of the anvil in preserving the left colic artery in laparoscopic low anterior rectal resection without auxiliary incisions for transanal specimen retrieval in this research.

**Methods:**

Clinical data and follow-up data of patients undergoing laparoscopic low anterior rectal resection from January 2017 to October 2020 were collected. The resections were modified such that the resisting nail holder was extracorporeally placed for the transanal exenteration of the specimen without using auxiliary abdominal incisions while preserving the left colic artery. By analyzing the data of anastomotic stenosis, anastomotic bleeding and anastomotic fistulas after surgery, the advantages and disadvantages of this surgical method for patients were clarified.

**Results:**

A total of 22 patients were enrolled. Five of 22 patients simultaneously underwent double-barrel terminal ileostomy. The postoperative exhaust time was 2–7 (median, 3) days. Postoperative anastomotic bleeding occurred in one patient, postoperative anastomotic fistula occurred in four patients, and postoperative anastomotic stenosis occurred in six patients. There were four patients with postoperative distant metastasis, of which three had concomitant local recurrence. Seventeen patients had no obvious symptoms or signs of recurrent metastases during follow-up appointments, and one died of liver failure.

**Conclusions:**

Modified laparoscopic low anterior rectal resection, which resects the specimen through anus eversion by inserting the anvil extracorporeally while preserving the left colic artery, is safe and feasible for patients with low rectal cancer.

## Background

Natural orifice specimen extraction surgery (NOSES) has become a prominent topic in the field of abdominal surgery, especially colorectal surgery, in recent years [1]. NOSES is a minimally invasive surgical procedure for rectal cancer. Its characterized by natural luminal specimens and complete intraperitoneal reconstruction of the digestive tract. NOSES refers to the type of surgery that uses laparoscopy, robots, transanal endoscopic microsurgery (TEM) or soft endoscopy and other equipment platforms to complete various routine surgical procedures (namely, resection and reconstruction) in the abdominal and pelvic cavity to extract specimens through a natural orifice of the human body (rectal, vaginal or oral cavity) without auxiliary incisions on the abdominal wall [2]. To date, the theoretical and technical system of NOSES related to colorectal tumours and the key points of operation techniques is becoming increasingly mature and standardised. Among them, laparoscopic low anterior rectal resection, in which the specimen is extracted transanally without auxiliary abdominal incisions (CRC-NOSES I), is the most widely used [3]. However, most recent reports about NOSES involve inserting the anastomotic anvil through the anus (rectus) or through an incision on the intestinal tube in the abdominal cavity or passing the anastomotic anvil through the intestinal tube. These processes inevitably result in abdominal infection [4]. Therefore, this study explored the surgical method of pulling the proximal intestine out of the body through the rectum before placing the anastomotic anvil to avoid intra-abdominal operations. Moreover, since pulling the proximal intestine out of the body requires fully freeing the proximal intestine while maintaining the blood supply, our study investigated the use of laparoscopic low anterior rectal resection, which resects the specimen through anal eversion by inserting the anvil outside the body while preserving the left colic artery for low rectal cancer.

## Methods

The inclusion criteria were as follows: (1) patients with low- to intermediate-grade rectal cancer; (2) tumour infiltration half of the circumference at most; (3) patients with no underlying diseases and (4) preoperative imaging was suggestive of T_1**–**3_N_0_M_0_. The exclusion criteria were as follows: (1) body mass index of > 30 kg/m^2^; (2) those with obstruction, perforation or bleeding, which urgently required surgery; (3) those receiving neoadjuvant treatment; (4) patients with a tumour whose maximum diameter is > 6 cm; (5) those with tumour with distant metastases, and (6) those sigmoid mesenteries were not enough to be pulled out through the rectum.

Preoperative determined the relative position of the inferior mesenteric artery (IMA), left colic artery and sigmoid colic artery. All patients enrolled in this study were cleaned by oral laxatives before surgery, and no mechanical bowel preparation was performed. The operation position, anesthesia procedure and trocar layout were the same as traditional operation. During the operation, the inflatable water injection test was performed to confirm that the anastomosis was unobstructed and that there was no leakage or bleeding. A protective terminal ileostomy was performed if there was a gas leak.

### Surgical procedure

**Fig. 2:**
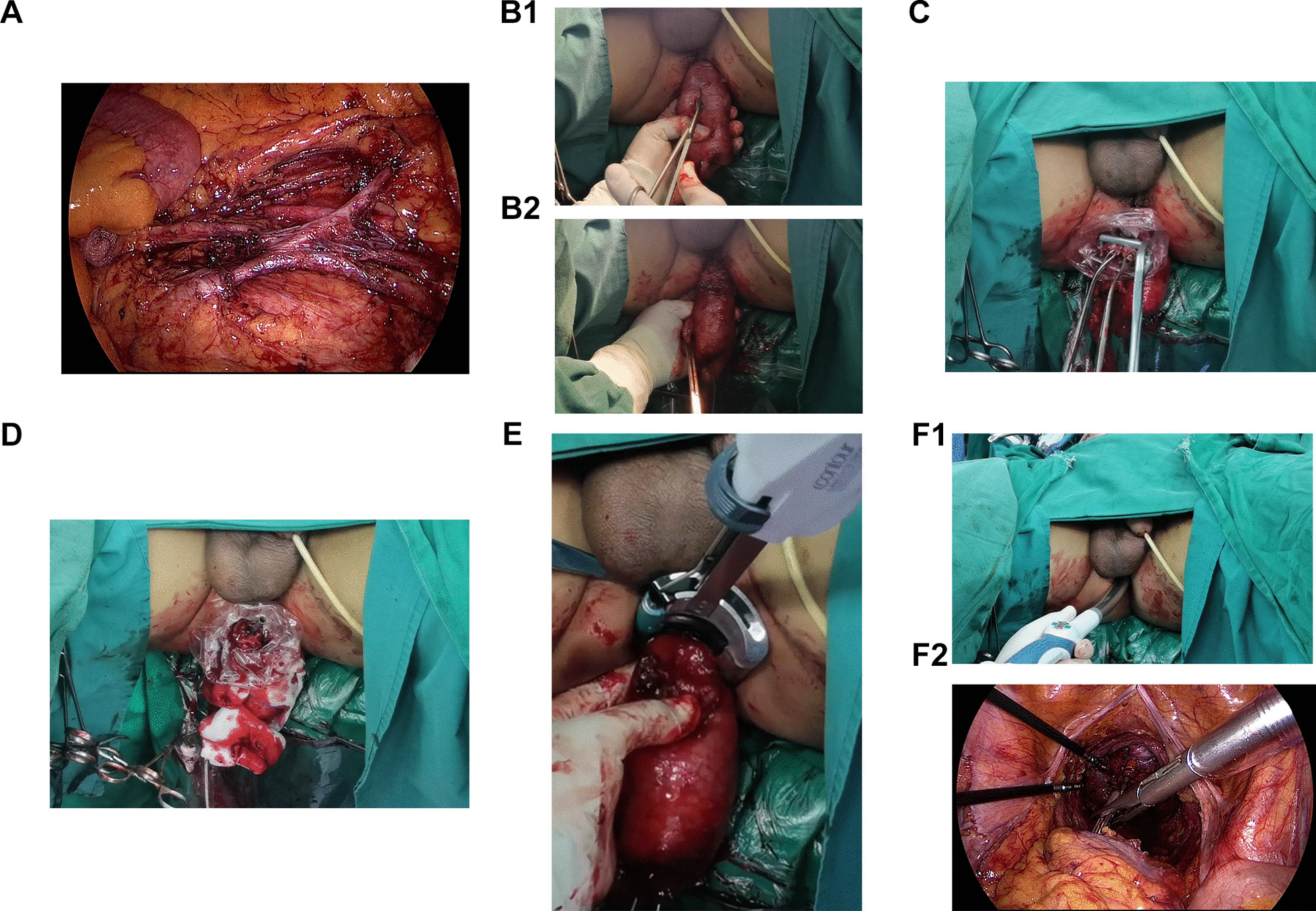
Operation steps of laparoscopic low anterior rectal resection (CRC-NOSES I), in which specimens are extracted through the anus without auxiliary abdominal incisions. Picture 2 is an actual picture of Picture 1. **a** The left colic vessels were exposed and the lymph nodes were dissected; **b** the specimen was everted in two steps; **c** purse-string forceps were used to clamp the proximal colon and create a purse-string suture; **d** the anvil was inserted; **e** the intestinal tube was disconnected at the distal end of the tumour; **f** the anastomat was inserted through the anus and an end-to-end anastomosis of the rectosigmoid colon was performed. The ownership of all pictures belongs to the author
- A two-step method was used to evert the specimen (Figs. 1b and 2b)
- Inserting the anastomotic anvil outside the body: the anvil was then inserted, and a knot was tied. After flushing with iodophor water, the proximal end of the sigmoid colon was placedback into the abdominal cavity (Figs. 1c, d and 2c, d).
- Disconnecting the rectum and the specimen was removed (Figs. 1e and 2e). Next, a tubular anastomat was inserted through the anus to complete the rectosigmoid colon end-to-end anastomosis (Figs. 1f and 2f).

- The 253 lymph node dissection and left colic artery preservation (Figs. 1a and 2a). Anterior rectal resection under the principle of TME. Disconnecting the proximal intestine.
  **Fig. 1:**
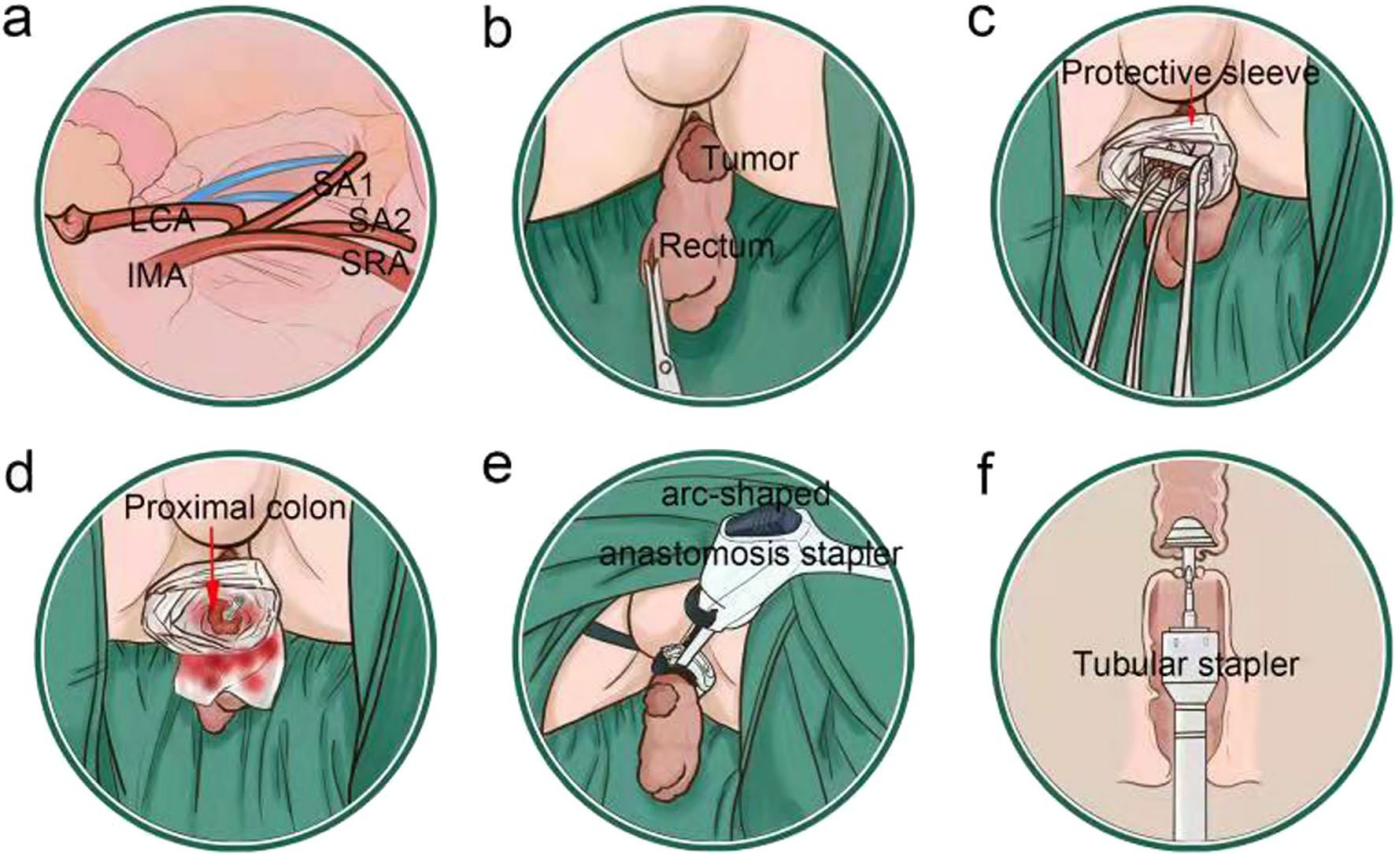
Operation steps of laparoscopic low anterior rectal resection (CRC-NOSES I), in which specimens are extracted through the anus without auxiliary abdominal incisions. **a** The left colic vessels were exposed and the lymph nodes were dissected; **b** the specimen was everted in two steps; **c** purse-string forceps were used to clamp the proximal colon and create a purse-string suture; **d** the anvil was inserted; **e** the intestinal tube was disconnected at the distal end of the tumour; **f** the anastomat was inserted through the anus and an end-to-end anastomosis of the rectosigmoid colon was performed. The ownership of all pictures belongs to the author

### Statistical analysis

This study is a descriptive study. All the statistical data will be discussed in a descriptive analysis and reflected in the Table 1.

**Table 1:** Data we collected from patients

Male/female	10/22
BMI, kg/m^2^ (median)	24.83
Age, years (median)	59
The lower edge of the tumour, cm (median)	4
Tumor location, n	22
Anterior wall	7
Posterior wall	5
Left lateral wall	3
Right lateral wall	7
Hospitalization time, days (median)	16
Intraoperative blood, ml (median)	20
The postoperative pathological staging, n	22
Stage I	7
Stage II	1
Stage III	14
The postoperative exhaust time, days (median)	3
Complications	
Anastomotic bleeding, n	1
Anastomotic fistulas, n	4
Anastomotic stenosis, n	6
Distant metastasis, n	4

## Results

We enrolled 22 patients who met the criteria mentioned above from January 2017 to October 2020 in the Department of Colorectal Surgery of the First Affiliated Hospital of Shandong First Medical University, including 10 men and 12 women whose median age was 59 (39–82) years and whose median body mass was 24.83 (19.8–29.2) kg/m^2^. The median lower edge of the tumour in each patient was 4 (3–9) cm from the anal edge. The preoperative staging was as follows: stage I in one case, stage II in six cases, and stage III in 15 cases. Tumours were located in the anterior wall of the rectum in seven cases, in the posterior wall in five cases, in the wall at the right side in three cases, and in the wall at the left side in seven cases. The tumour infiltrated one-fifth to one-half of the intestinal lunmen. The median length of hospitalisation was 16 (11–36) days. The median follow-up period was 18 months. All the patients underwent laparoscopic anterior rectal resection (Table 1).

The median intraoperative blood loss was 20 (20–100) ml, whereas the postoperative pathological staging was as follows: stage I in seven cases, stage II in one case and stage III in 14 cases. Wherein, T staging was as follows: T1 in one case, T2 in twelve cases and T3 in 9 cases. The median postoperative exhaust time was 3 (2–7) days. We graded postoperative complications according to the Clavien-Dindo classification. Postoperative anastomotic bleeding (stage IIIa) occurred in one patient, which was relieved via haemostasis under endoscopy. Four patients developed postoperative anastomotic fistulas. In three of them (stage II), the fistulas were relieved via fasting after surgery, and the remaining one (stage IIIb) underwent a transverse colonic single-barrel stoma. Anastomotic fistulas were found in three patients by electronic colonoscopy during follow-up. One patient developed opacity from pelvic drainage fluid after the operation, which proved to be an anastomotic fistula after a second operation. Six patients had postoperative anastomotic stenosis, five (stage IIIa) underwent TEM surgery to loosen the stenosis ring, and one (stage I) had anal dilatation; all of the symptoms subsided. There were four cases of postoperative distant metastasis (all were postoperative pathological stage III), of which three had concomitant local recurrence. There were no obvious signs of recurrence or metastasis in 17 patients during their follow-up appointments, and one patient died of liver failure.

## Discussions

NOSES is a minimally invasive surgical method first proposed and summarised systematically by Professor Wang Xishan in China [4]. Natural orifice specimen extraction is the most distinctive core content of NOSES, and it is the most concerning and prominently discussed operation link. Due to the differences in tumour location and size and the differences in surgeons’ experiences, the operation process of natural orifice specimen extraction also reflects strong individual differences. With the deepening of the understanding of the theoretical system of NOSES, the NOSES I for low rectal cancer has been perfected in six operation modes [5].

For the NOSES operation for low rectal cancer, while pursuing minimal invasiveness and preservation of the anus, first, it is necessary to have sufficient distal margins to ensure the safety of the tumour. Second, complications related to the operation and anastomosis, such as anastomotic leakage, anastomotic stenosis and abdominal infection, should be avoided as much as possible. In this study, one patient had blood in the stool on the sixth day after surgery. It was found under colonoscopy that the bleeding originated from haemorrhoids. Symptomatic treatment was given, and the blood in the stool subsided. This symptom may have been due to the haemorrhoids being stimulated by the anastomat during the operation and the postoperative treatment with anticoagulant drugs. Another patient developed fever and abdominal pain on the third day after surgery.

Anastomotic fistula is one of the complications after colorectal surgery. It not only increases the risk of abdominal infection in patients but also has a great impact on the prognosis of patients. The occurrence of an anastomotic fistula is related to the distance between the anastomosis and the anal margin. The lower the anastomotic stoma, the higher the incidence, which can reach up to 20.6% if the distance is within 5 cm [6–9]. We believe that the key to preventing anastomotic fistula is to ensure a tension-free state and sufficient blood supply to the anastomotic stoma during the operation. Observing the colour of the drainage fluid in the early stage of the operation is also helpful for the early detection and diagnosis of anastomotic fistula.

In this study, there were three patients with anastomotic fistulas without obvious clinical symptoms. Colonoscopy, repeated 6 months after the operation, revealed a small fistula around the anastomotic stoma with anastomotic stenosis. The cause of the stenosis may be the delayed primary healing of the anastomosis, the hyperplasia and fibrosis of the granulation tissue and the cicatricial stenosis. Our preferred treatment for anastomotic stenosis is multiple finger dilations. Five patients with poor results underwent TEM surgery to loosen the stenosis ring, and the lithotomy positions at 6 and 9 o’clock were used as the incision positions [10]. The national and global rate of stenosis of the anastomotic stoma after colorectal surgery can reach 21.1% [11, 12]; thus, preventing anastomotic stenosis is particularly important. During the operation, the blood supply of the arterial arch at the edge of the intestines and the relaxation of the intestinal tension after anastomosis should be ensured. Four weeks after the surgery, regular finger dilation, enema and other rehabilitation techniques should be performed [13]. There were four cases of postoperative distant metastasis (all were postoperative pathological stage III), of which three had concomitant local recurrence. One patient underwent lobectomy surgery after finding pulmonary metastasis, and the prognosis was good. Two patients received radiotherapy and chemotherapy. One patient finally chose to give up treatment due to family problems. Some studies have shown that preserving the left colonic artery does not increase the overall recurrence rate and reduce the overall survival rate, and there is no significant difference in long-term survival [14, 15].

In this study, our anvil insertion method completely prevented the contamination of the abdominal cavity by intestinal cavity content or bacteria that might be caused by the incision of the intestinal tube or the insertion of the anvil in the abdominal cavity. Moreover, under the protection of the laparoscopic protective sleeve, contact with the tumour tissue was also avoided. The purse-string suture of the proximal intestinal tube also avoided the dangerous angle formed by the closing of the laparoscopic anastomosis stapler and reduced the incidence of potential anastomotic leakage. In addition, due to the application of purse-string forceps, the number of laparoscopic anastomosis staplers required was reduced, which lessened medical expenses.

However, because this surgical method required the proximal intestinal tube to be pulled 3–5 cm outside the anus, it was necessary to free it fully. At this time, there were two problems: (1) high ligation of the inferior mesenteric artery could obtain a longer free intestinal tube [16], but the distal blood supply was relatively poor [17]; (2) keeping the left colic artery could ensure adequate blood supply, but the conventional reservation of the left colic artery would increase the mesangial tension, which might make it impossible to pull the intestinal tube out of the body. Bonnet et al. found, via autopsy, that high ligation of IMA could obtain 10 cm more of free intestines than low ligation. However, the data obtained in the autopsy may have certain limitations in clinical application [18].

Based on the understanding of membrane anatomy and mesangial theory and the summarised experience in laparoscopic left colectomy, our team believed that there are two causes for the tension of the descending colon and sigmoid colon: (1) the presence of the splenic flexure, including the gastrocolonic ligaments and splenic colon ligament and adhesion between the descending colon and the lateral abdominal wall may contribute to the tension; therefore, a certain length of the free intestine can be obtained by loosening the splenic flexure and freeing the adhesion between the descending colon and the lateral abdominal wall; (2) the natural fusion of the left mesentery of the transverse colon with the dorsal mesentery and mesangial bed of the descending colon, wherein the left colic artery runs in the left mesentery of the transverse colon and the mesentery of descending colon, may also be a contributing factor. Therefore, under the premise that the inferior mesenteric vein is disconnected at a high position after the mesentery of the descending colon and the left mesentery of the transverse colon are fully freed, the remaining left colic artery may no longer form traction and tension on the intestinal tubes. This study also confirmed our view that in all the patients in whom the left colic artery is retained, freeing the splenic flexure and the mesentery of the descending colon provided a sufficient length of proximal intestinal tubes to ensure that the anastomotic stoma is tension-free.

The advantages of this surgical method are as follows: (1) the anvil is completely inserted outside the abdominal cavity, which reduces the possibility of abdominal infection [19]; (2) the digestive tract reconstruction process avoids tumour contact; (3) the rectum is dissected outside the body under direct vision, which ensures negative surgical margins of the tumour; (4) the left colic artery is preserved, tension-free anastomosis is achieved, and the occurrence of anastomotic leakage is reduced [20, 21]; (5) anal preservation for low rectal cancer is realised; and (6) medical expenses are lessened.

This surgical method was only retrospectively study and carried out by a single centre. Therefore, the details and quality of the operation need to be improved, and the data related to long-term survival are still being followed up and collated.

## Conclusions

Our modified surgical approach is safe and effective and does not harm the interests of patients. As reported by Expert Consensus on NOSES for Colorectal Tumours inclusion criteria are crucial to perform this procedure.

## Data Availability

All data generated or analysed during this study are included in this published article. All methods were carried out in accordance with relevant guidelines and regulations.

## Abbreviations

NOSES: Natural orifice specimen extraction surgery
TEM: Transanal endoscopic microsurgery
CRC-NOSES I: Laparoscopic low anterior rectal resection, in which the specimen is extracted transanally without auxiliary abdominal incisions
IMA: The inferior mesenteric artery
LCA: Left colic artery
SCA: Sigmoid colic artery
